# Supplementary material for: Pork Fat and Meat: A Balance between Consumer Expectations and Nutrient Composition of Four Pig Breeds
Source: Foods. 2023 Feb 5;12(4):690. doi: 10.3390/foods12040690 (PMC9955543; doi:10.3390/foods12040690)
Supplement: Supplementary file 1 [file foods-12-00690-s001.zip › Supplementary Files.pdf]

Table S1. Area and intensity of Raman spectra peaks of pork backfat

Pig breeds	Output	Peak, sm^-1				
		960-980	1260-1280	1290-1310	1420-1470	1720-1760
A	Area	32863±6491^{a}	94009±28975^{a}	238663±8643	868534±143950	89433±44072
	Intensity	2958±366^{a}	11524±1157^{a}	19052±932	30236±28	4572±235^{a}
D	Area	14867±8002^{b}	61144±60945	191120±7646^{a}	754474±464776	83968±38212
	Intensity	2959±1516^{a}	9817±2824^{c}	18673±2463^{a}	31093±1852	3846±476^{b}
L	Area	16408±1133^{b}	26789±7738^{b}	278295±7573^{b}	914833±158254	106970±644
	Intensity	1355±29^{b}	6951±189^{b,d}	20999±773^{b}	30185±232	4174±99
M	Area	21309±3231^{b}	62617±22774	250018±1174^{b}	765778±176441	108650±5027
	Intensity	1755±248^{b}	8324±761^{b}	19272±786	30154±198	4218±217

* a-b, c-d– Different letters indicate statistically significant differences (p≤0.05)
